# Combined effects of niclosamide and temozolomide against human glioblastoma tumorspheres

**DOI:** 10.1007/s00432-020-03330-7

**Published:** 2020-07-25

**Authors:** Hyeong-Cheol Oh, Jin-Kyoung Shim, Junseong Park, Ji-Hyun Lee, Ran Joo Choi, Nam Hee Kim, Hyun Sil Kim, Ju Hyung Moon, Eui Hyun Kim, Jong Hee Chang, Jong In Yook, Seok-Gu Kang

**Affiliations:** 1 Department of Neurosurgery, Brain Tumor Center, Severance Hospital, Yonsei University College of Medicine, 50-1 Yonsei-ro, Seodaemun-gu, Seoul, 03722 Republic of Korea; 2 Department of Oral Pathology, Yonsei University College of Dentistry, 50-1 Yonsei-ro, Seodaemun-gu, Seoul, 03722 Republic of Korea; 3 Department of Medical Science, Yonsei University Graduate School, 50-1 Yonsei-ro, Seodaemun-gu, Seoul, 03722 Republic of Korea

**Keywords:** Glioblastoma, Invasion, Niclosamide, Temozolomide, Tumorsphere

## Abstract

**Purpose:**

Glioblastoma (GBM) is the most aggressive type of brain tumor and has poor survival outcomes, even after a combination of surgery, radiotherapy, and chemotherapy. Temozolomide is the only agent that has been shown to be effective against GBM, suggesting that combination of temozolomide with other agents may be more effective. Niclosamide, an FDA approved anthelmintic agent, has shown anti-cancer effects against human colon, breast, prostate cancers as well as GBM. However, the efficacy of the combination of niclosamide with temozolomide against GBM tumorspheres (TSs) has not been determined. We hypothesized that the combined treatment could effectively suppress GBM TSs.

**Methods:**

GBM TSs (TS15-88, GSC11) were treated with niclosamide and/or temozolomide. Combined effects of two drugs were evaluated by measuring viability, neurosphere formation, and 3D-invasion in collagen matrix. Transcriptional profiles of GBM TS were analyzed using RNA sequencing. In vivo anticancer efficacy of combined drugs was tested in a mouse orthotopic xenograft model.

**Results:**

Combination treatment of niclosamide and temozolomide significantly inhibited the cell viability, stemness, and invasive properties of GBM TSs. This combined treatment significantly down-regulated the expression of epithelial mesenchymal transition-related markers, Zeb1, *N*-cadherin, and *β*-catenin. The combined treatment also significantly decreased tumor growth in orthotopic xenograft models.

**Conclusion:**

The combination of niclosamide and temozolomide effectively decreased the stemness and invasive properties of GBM TSs, suggesting that this regimen may be therapeutically effective in treating patients with GBM.

**Electronic supplementary material:**

The online version of this article (10.1007/s00432-020-03330-7) contains supplementary material, which is available to authorized users.

## Introduction

Patients with glioblastoma (GBM), the most common type of primary brain tumor, have a poor prognosis, with a median overall survival of about 17.5 months in Korea (Kim et al. 2017a). Standard treatments in patients with GBM include chemotherapy with the alkylating agent temozolomide, along with surgical resection and radiotherapy (Roh et al.  2017, 2020; Stupp et al. 2005). Outcomes may be improved by combining temozolomide with other therapeutic agents, especially old or failed drugs (Chong and Sullivan 2007). Because the pharmacokinetics of existing drugs have been determined, and because many of these agents have been approved for human use, many clinical trials have evaluated new uses of old drugs (Chong and Sullivan 2007).

Niclosamide is an anthelmintic agent approved by the U.S. Food and Drug Administration (FDA) that has been used to treat tapeworm infection for approximately 50 years (Garin et al. 1964). Recent drug screening has identified niclosamide as a potential anticancer agent (Li et al. 2014). This agent has been found to inhibit cancer-associated signaling cascades, including Wnt (Sack et al. 2011), mTORC1 (Balgi et al. 2009), STAT3 (Ren et al. 2010), NF-κB (Wieland et al. 2013) and Notch (Wang et al. 2009). In addition, the anti-cancer effects of niclosamide have been demonstrated in human colon (Sack et al. 2011), breast (Londono-Joshi et al. 2014), prostate (Lu et al. 2011), lung (Stewart et al. 2016) cancers as well as in GBM (Wieland et al. (2013); Cheng et al. 2017).

GBM tumorspheres (TSs) are cells refractory to treatment (Kang et al. 2015; Kong et al. 2013a, b), making them a good testing platform to evaluate the efficacy of drugs (Kim et al. 2017b; Choi et al. 2016; Park et al. 2018). Niclosamide was found to have anticancer effects against GBM TSs in vitro (Wieland et al. 2013; Cheng et al. 2017) and in vivo (Wieland et al. 2013). However, the effects of the combination of niclosamide and temozolomide on GBM TSs in vivo have not yet been determined. In this study, we examined combined effects of niclosamide and temozolomide using patient-derived TSs and a mouse orthotopic xenograft model. Our findings suggest that this combined treatment could be a new treatment option for GBM.

## Materials and methods

### Cell culture and reagents

Two primary tumor cells derived from GBM patients, TS15-88 and GSC11, were used to create TS models in this study. TS15-88 was established from fresh GBM tissue specimens, as approved by the institutional review board of Yonsei University College of Medicine (4-2014-0649). Patient-derived GSC11 cells were provided by Dr. Frederick F. Lang (Department of Neurosurgery, The University of Texas, M. D. Anderson Cancer Center, Houston, Texas, USA). For TS culture, cells were cultured in TS complete media composed of DMEM/F-12 (Mediatech, Manassas, VA, USA), 1 × B27 (Invitrogen, San Diego, CA, USA), 20 ng/ml of bFGF, and 20 ng/ml of EGF (Sigma-Aldrich, St. Louis, MO, USA) (Kwak et al. 2013). All in vitro experiments were performed under TS culture conditions. For in vitro treatments, niclosamide and TMZ were dissolved in DMSO and added to cell cultures to the desired concentration.

### Characterization of GBM TSs

TS formation from human GBM specimens followed previous methods (Kong et al. 2013b), and their expression of stemness markers, CD133, nestin, musashi, and podoplanin (Abcam, Cambridge, UK), was tested by immunocytochemistry. Neuroglial differentiation in GBM TSs was evaluated by monitoring the expression of GFAP (Dako, Carpinteria, CA, USA), MBP, NeuN, and TUBB3 (Chemicon, Temecula, CA, USA).

### Cell viability assay

The effects of niclosamide, TMZ, and the combination of niclosamide and TMZ on cell survival were determined using MTS viability assays (Promega, Madison, WI, USA) (Mosmann 1983). GBM TS cells seeded in 96-well plates (1 × 10^4^ cells/well) were incubated at 37 °C for 24 h and treated with niclosamide and/or temozolomide for 3 days. MTS reagent (20 μl/well) was added, the cells were incubated at 37 °C for 2 h, and the absorbance of each well was measured at 595 nm. Each experiment was repeated three times in triplicate, with the results expressed as the percentage of viable cells relative to controls. Synergy score for combination treatment of niclosamide and TMZ was calculated using Bliss method. Combination indices (CIs) of combined treatment with niclosamide and temozolomide were calculated by CompuSyn software.

### Sphere formation assay

Dissociated 10 single GBM TSs were cultured in 96-well plates in medium containing DMEM/F-12 (Mediatech, Manassas, VA, USA), supplemented with 1 × B27 (Invitrogen, San Diego, CA, USA), 20 ng/ml of bFGF, 20 ng/ml of EGF (Sigma-Aldrich, St. Louis, MO, USA), and 50 U/ml penicillin/50 mg/ml streptomycin. After 3 weeks of incubation under different conditions, the number of sphere-positive wells was counted, and the proportion of sphere-positive wells in the treatment group relative to that in controls was calculated and presented as a percentage. Images of sphere positive wells were captured and analyzed using ToupView software (ToupTek Photonics, Zhejiang, China).

### Invasion assay

GSC-11 and TS15-88 cells grown as single spheroids were seeded and cultured in individual wells of a 96-well plate. Each well was filled with mixed matrix composed of Matrigel, collagen type I (Corning), and TS complete media. Single spheroids were seeded inside the matrix prior to gelation. Then, TS complete media was added over the gelled matrix to prevent drying. The invaded area was quantified as occupied area at (72–0 h)/0 h.

### Western blot analyses

Cell lysates were separated by SDS-PAGE on 10% Tris–glycine gels. Proteins were transferred to nitrocellulose membranes and probed with antibodies against Sox2 (Merck Millipore, Billerica, MA, USA); Nestin (Novus Biologicals, Littleton, CO, USA); PDPN and *β*-catenin (Cell Signaling Technology, Beverly, MA, USA); *N*-cadherin (R&D Systems); Zeb1 (Sigma-Aldrich); STAT3 (Cell Signaling Technology); MGMT (MT3.1); Phosphorylated STAT3 (Cell Signaling Technology); and GAPDH (Santa Cruz Biotechnology, Santa Cruz, CA, USA). Proteins were detected using horseradish peroxidase-conjugated IgG (Santa Cruz Biotechnology) in conjunction with Western Lightning Plus-enhanced chemiluminescence reagent (PerkinElmer, Waltham, MA, USA). Images were captured using an ImageQuant LAS 4000 mini (GE Healthcare Life Sciences, Little Chalfont, UK).

### RNA QC, library construction, and sequencing

The quality and quantity of total RNA were assessed by Agilent 2100 Bioanalyzer with a Eukaryotic Total RNA Pico chip (Agilent Technologies). Libraries were quantified using the Agilent TapeStation 4200 HSD1000 screen tapes (Agilent Technologies) and KAPA Library Quantification Kit (KK4824, Kapa Biosystems). The individual samples were pooled and sequenced on the Illumina NovaSeq6000 with 150 bp paired-end by following the manufacturer's protocols. Image analysis were performed using the NovaSeq6000 control Software version 1.3.1 and the output data was demultiplexed with bcl2fastq v2.2 generating fastqc files. Detailed description of experimental materials and methods is given in the supplementary experimental procedures.

### Preprocessing of transcriptome data

The quality of the reads was checked using fastQC (v.0.10.1) and the sequencing adapters were removed using trimmomatic (v. 0.38). Low quality reads were filtered according to the following criteria; reads contain more than 10% of skipped bases (marked as ‘N’s), reads contain more than 40% of bases whose quality scores are less than 20, and reads whose average quality scores of each read are less than 20. Filtered reads were mapped to the human reference genome (Ensembl release 72 (Flicek et al. 2013)) using the aligner Tophat (Trapnell et al. 2009). Gene expression level was measured with Cufflinks v2.1.1 (Trapnell et al. 2012) using the gene annotation database of Ensembl release 72. Non-coding region was removed with—mask option.

### Functional annotation to DEGs

A total of 1391 DEGs (One-way ANOVA with Tukey’s post hoc test; *P* < 0.001) was identified between control and combination groups. Functional annotation to these DEGs was performed by over-representation analysis (ORA) using GO gene sets, and then visualized as an enrichment map using Cytoscape (Shannon et al. 2003) and ClueGO (Bindea et al. 2009) plug-in. Enriched GO terms were functionally categorized based on their kappa scores (> 0.4). Statistical significance was determined using two-sided hypergeometric test, and only nodes with Bonferroni-adjusted *P* value < 0.05 were displayed.

### Mouse orthotopic xenograft model

Male athymic nude mice (4–8 weeks old; Central Lab. Animal Inc., Seoul, Korea) were used in this study. Mice were housed in micro-isolator cages under sterile conditions and monitored for at least 1 week before study initiation to ensure proper health. Lighting, temperature, and humidity were controlled centrally. Mice were anesthetized with a solution of Zoletil (30 mg/kg; Virbac Korea, Seoul, Korea) and xylazine (10 mg/kg; Bayer Korea, Seoul, Korea), which was administered intraperitoneally. GBM TSs (GSC11) were pretreated by niclosamide (500 nM), TMZ (250 µM), and combination of niclosamide and TMZ for 3 days with reference to the pretreatment method from several studies (Liu et al. 2016; Natale et al. 2018; Wang et al. 2019; Xia et al. 2017). DMSO-control (*n* = 5), niclosamide (*n* = 5), TMZ (*n* = 5), and combination of niclosamide and TMZ (*n* = 5)—pretreated GBM TSs were implanted into the right frontal lobe of nude mice using a guide-screw system (Lal et al. 2000). total of 5 × 10^5^ cells was injected to a depth of 4.5 mm using a Hamilton syringe (Dongwoo Science Co., Seoul, Korea). Mice were euthanized according to the approved protocol if daily monitored body weight had decreased by more than 15% compared to the original body weight.

### Bioluminescence imaging

Bioluminescence acquisition and analyses were performed using an IVIS imaging system and Living Image v4.2 software (Caliper Life Sciences, Hopkinton, MA, USA). Mice were injected intraperitoneally with 100 μl d-luciferin (30 mg/ml; Promega) 15 min prior to signal acquisition (5 s), which took place under 2.5% isoflurane anesthesia. Grayscale photographic images and bioluminescence color images were superimposed.

### Statistical analysis

Levels of significance for comparisons among treatment groups were determined using one-way ANOVA with Tukey’s post hoc test for multiple comparisons. Bliss expectation was calculated as (A + B) – A × B, where A and B are the fractional growth inhibitions of drug A and B at a given dose. Survival analysis was performed using Kaplan–Meier curves with log-rank test. Results were considered statistically significant at a *P* value < 0.05. GraphPad Prism 6 (GraphPad Software Inc.) was used for quantitative analysis.

## Results

### Characterization of GBM TSs

Morphologically, GSC11 and TS15-88 cells cultured in TS complete media were spheroid shaped. Immunocytochemistry showed that both GSC11 and TS15-88 TSs were positive for the stem cell markers, CD133 and Nestin, whereas only GSC11 TSs were further positive for Podoplanin (PDPN) and Musashi. Neuroglial differentiation was successfully induced in all TSs, which was confirmed by positive GFAP, MBP, NeuN and TUBB3 stains (Supplementary Fig. 1).

### Combined treatment with niclosamide and TMZ synergistically reduces cellular viability of GBM TSs

**Fig. 1:**
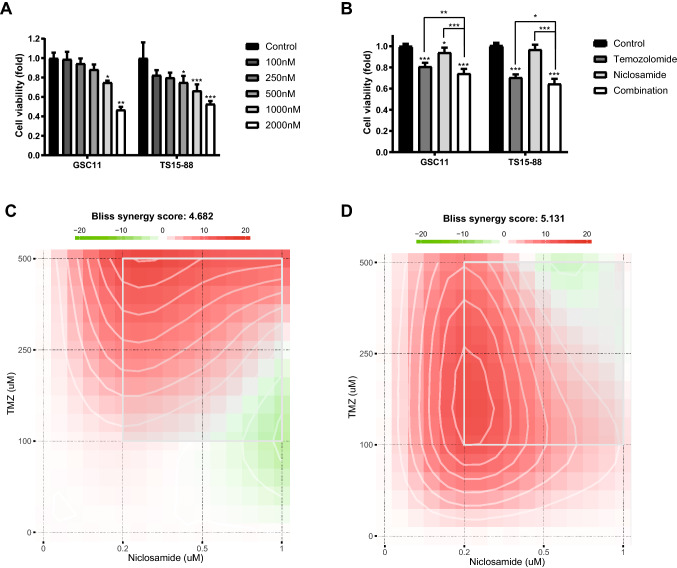
Effects of combined treatment with niclosamide and TMZ on the viability of GBM TSs.** a** The viability of GBM TSs was measured 72 h after treatment with niclosamide alone at different concentration. **b** Cell viability was measured 72 h after treatment with niclosamide and/or TMZ. The drug interaction landscapes based on the bliss model. The bliss synergy score of drug combination with niclosamide and TMZ against **c** GSC11 **d** TS15-88 is shown. Differences among groups were compared by one-way ANOVA with Tukey’s post hoc test; means ± SD; *P < 0.05, ***P < 0.001, where asterisks denote significant differences between the indicated groups or compared with controls (asterisks over the bar)

To determine an optimal working concentration of niclosamide, its cytotoxicity against GBM TSs was tested. Treatment of both GSC11 and TS15-88 TSs with niclosamide reduced cell viability in a concentration dependent manner (Fig. 1a). MTS assays testing the viability of GBM TS cells treated with niclosamide (500 nM), and/or temozolomide (250 μM) for 3 days showed that the combination treatment of niclosamide and TMZ more effectively inhibited the proliferation of GBM TSs than either agent alone or untreated controls (Fig. 1b). The synergy scores are calculated across all the tested concentration combinations by bliss method, showing that combined treatment of niclosamide and TMZ synergistically inhibited both GBM TSs (GSC11 and TS15-88) (Fig. 1c, d). The combination index of combined treatment of niclosamide (500 nM) and TMZ (250 μM) is shown in supplementary Fig. 2, suggesting an existence of synergism between the drugs.

**Fig. 2:**
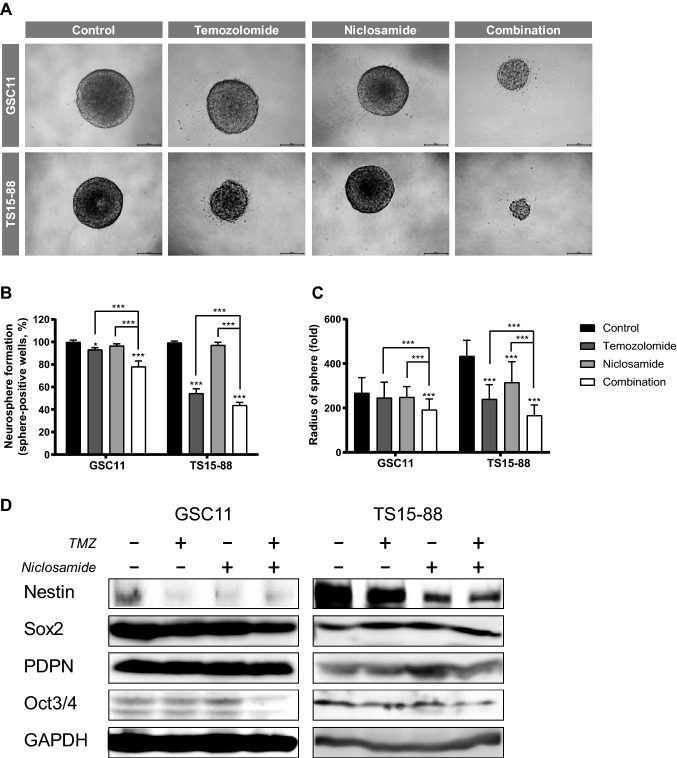
Evaluation of stemness of GBM TSs after treatment with niclosamide and TMZ. **a** spheroid shaped GBM TSs. Stemness was determined by neurosphere formation assays, quantified as the percentage of **b** sphere-positive wells and **c** sphere radius. **d** Expression of genes related to stemness was measured by western blot analysis. Differences among groups were compared by one-way ANOVA with Tukey’s post hoc test; means ± SD; *P < 0.05, ***P < 0.001, where asterisks denote significant differences between the indicated groups or compared with controls (asterisks over the bar)

### Combined treatment with niclosamide and TMZ suppresses stemness of GBM TSs

Sphere-formation assays showed that temozolomide, niclosamide, and their combination differed in their ability to inhibit the stemness of GBM TSs. GBM TS morphology was more affected by combination treatment than by either agent alone (Fig. 2a). Although temozolomide alone somewhat reduced the proportion of sphere-positive wells and niclosamide alone was ineffective, niclosamide enhanced the anti-stemness activity of temozolomide (Fig. 2b), reducing the sphere radii (Fig. 2c). Western blot analyses showed that the combination of niclosamide and temozolomide reduced the expression of stemness-related proteins, including nestin and Oct3/4 (Fig. 2d).

### Combined treatment with niclosamide and TMZ suppresses invasiveness of GBM TSs

**Fig. 3:**
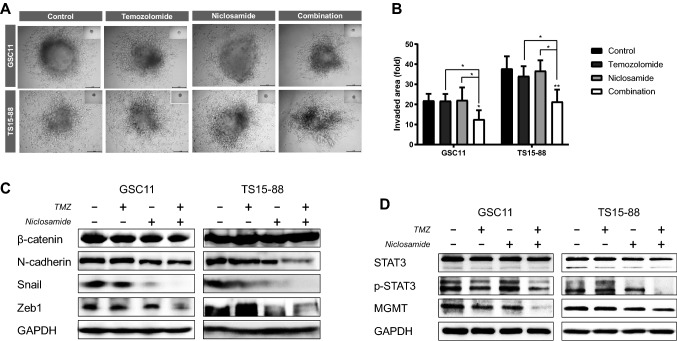
Effects of treatment with niclosamide and/or TMZ on the invasive properties of GBM TSs and expression of mesenchymal transition markers. Invasiveness were measured after 72 h of treatment with niclosamide and TMZ. **a** Decreased invasiveness of implanted GBM TSs was demonstrated using 3D collagen matrix invasion assays, under treatment with niclosamide and TMZ, alone and in combination (scale bar = 50 μM). **b** Inhibitory effects of drug treatment on invasiveness were quantified by measuring the area of invasion. **c** Expression of proteins related to mesenchymal transition and invasion was measured by western blot analysis. **d** Immunoblot analysis of MGMT, STAT3, and p-STAT3 in TSs treated with combination of niclosamide and temozolomide. Differences among groups were compared by one-way ANOVA with Tukey’s post hoc test; means ± SD; *P < 0.05, ***P < 0.001, where asterisks denote significant differences between the indicated groups or compared with controls (asterisks over the bar)

For 3D invasion assays, we implanted GFP-GBM TSs in a collagen type 1 matrix and evaluated the anti-invasion effects of niclosamide and/or temozolomide after 72 h. Invasion morphology was more influenced by combination treatment than by either temozolomide or niclosamide alone (Fig. 3a). Compared with untreated GBM TSs, the combination of niclosamide (500 nM) and temozolomide (250 μM) significantly inhibited invasion by the GBM TSs, GSC11 and TS15-88 cells (Fig. 3b). Western blot analyses showed that combined treatment with niclosamide and temozolomide of both TSs reduced the expression of the EMT-related markers, *N*-cadherin, Snail, and Zeb1 (Fig. 3c). The combination treatment of niclosamide and temozolomide also suppressed expressions of MGMT and phosphorylated STAT3 (Fig. 3d).

### Transcriptional profiles following niclosamide and/or TMZ treatment

**Fig. 4:**
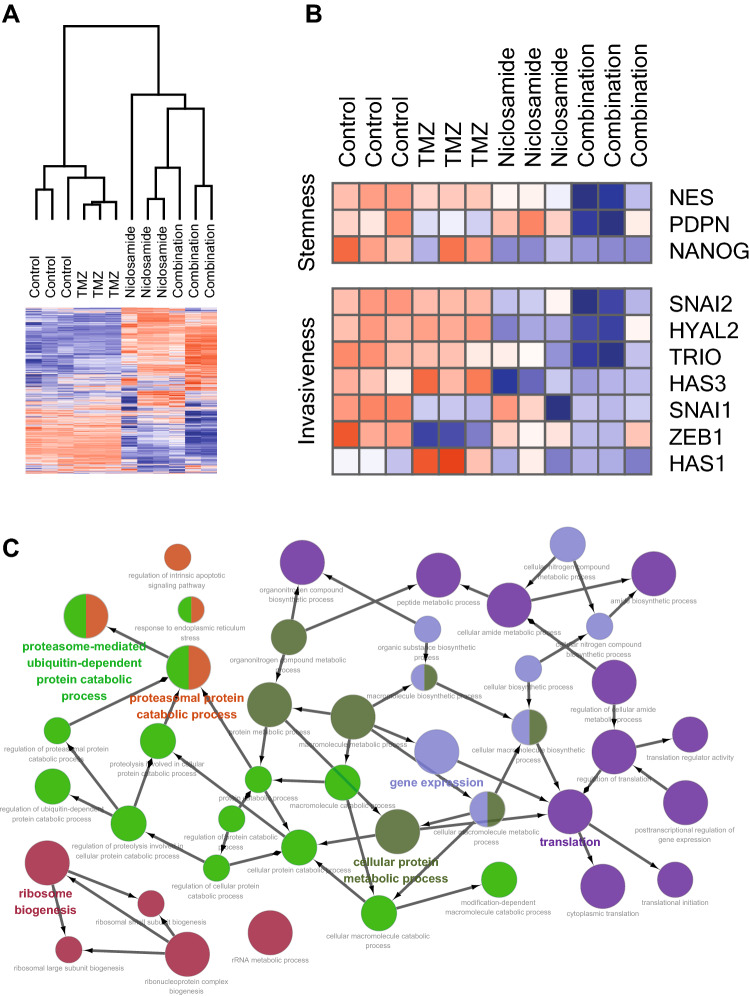
GSC11 cells were treated with niclosamide and TMZ alone or in combination for 72 h, and gene expression profile was obtained using RNA-sequencing. **a** For genes with average expression levels of top 30%, average linkage hierarchical clustering was performed with Euclidean distance as a distance metric, and expression levels were depicted as a heat map using GENE-E software. **b** Expression levels of stemness- and invasiveness-associated genes were displayed as a heat map. **c** Among 1391 DEGs between control and combination groups, 892 genes whose expression levels were upregulated in combination group were functionally annotated, clustered, and visualized as an enrichment map. Each node represents a GO term, with the node size reflecting statistical significance for over-representation. An edge between two nodes denotes kappa score relationship. Node colors reflect clustered modules; the most significant GO terms for each module have highlighted labels

We next used RNA sequencing to examine the effect of niclosamide and TMZ on transcriptional profiles of GBM TS (GCS11). Hierarchical clustering of differentially expressed genes (DEGs) with expression levels of top 30% showed strong intragroup clustering and distinct expression patterns compared with controls (Fig. 4a). A subset of genes, which encode proteins that regulate cell stemness and EMT such as NES (nestin), SNAI2 (TWIST), and ZEB1 were significantly downregulated, validating western-blotting data treated with combination of niclosamide and TMZ (Fig. 4b). Among 1391 DEGs from control and combination groups, 892 genes from combination group were upregulated. Functional annotation of these DEGs using Gene Ontology (GO) database revealed that protein catabolism and autophagy-related gene sets were exclusively enriched by combined therapy (Fig. 4c).

### Therapeutic responses in a mouse orthotopic xenograft model

**Fig. 5:**
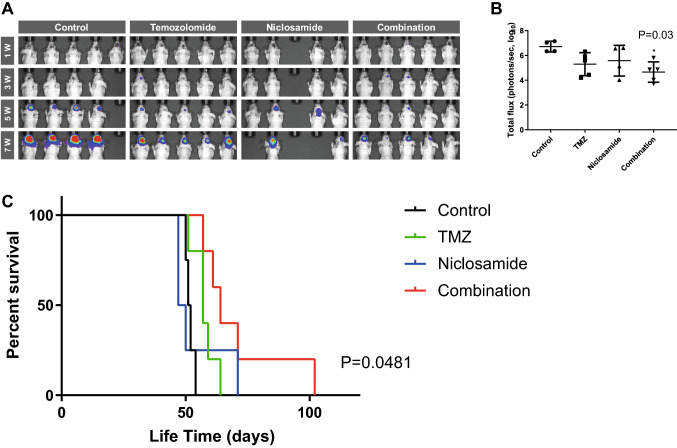
Therapeutic responses in a mouse orthotopic xenograft model. **a** Tumor volume was measured by bioluminescence imaging. **b** Signal intensity was quantified as total photon flux from tissues on the 5th week (*P < 0.05; one-way ANOVA with Tukey’s post hoc test). **c** Kaplan–Meier survival curve showed increased survival of mice treated with the combination of niclosamide and temozolomide compared to the control (*P* = 0.0481)

The in vivo therapeutic effects of niclosamide and/or temozolomide on tumor growth were analyzed in a mouse orthotopic xenograft model using GBM TSs (GSC11). Bioluminescence imaging showed that, compared with either agent alone, the combination of niclosamide and temozolomide significantly reduced the tumor burden compared to the control in this orthotopic xenograft model (Fig. 5a, b). In the Kaplan–Meier survival analysis, different anti-cancer effects were shown accordingly to treatment groups (Fig. 5c). Combined treatment with niclosamide and TMZ confers significant survival benefits compared to the control.

## Discussion

Despite recent progress in understanding the biology of GBM, many tested chemotherapy strategies have been found that they do not confer significant survival advantages over treatment with temozolomide alone. Accordingly, more effective therapeutic agents are required for clinical management of GBM. Because TSs derived from GBM are highly resistant to radiation and chemotherapy (D'Alessandris et al. 2017), we screened drug regimens against GBM TSs (Kim et al. 2017b; Choi et al. 2016; Park et al. 2018).

Although niclosamide has anti-cancer effects against various types of cancer including GBM (Sack et al. 2011; Wieland et al. 2013; Londono-Joshi et al. 2014; Lu et al. 2011; Stewart et al. 2016), the in vivo combination effects of niclosamide and TMZ against GBM TSs are still unknown. The present study evaluated the effects of treatment with niclosamide and temozolomide, alone and in combination, on the stemness and invasiveness of GBM TSs, demonstrating that combination treatment effectively reduced the stemness and invasive properties (Lim et al. 2020) of GBM TSs. Furthermore, we confirmed for the first time that the combination treatment of niclosamide and TMZ significantly prolong overall survival compared to the control group in a mouse orthotopic xenograft model.

Firstly, we found that niclosamide significantly reduced proliferation of GBM TSs in a dose dependent manner. Chien et al. (2018) reported that niclosamide inhibited invasiveness but could not alter cell viability in hepatocellular carcinoma. However, several studies have revealed that niclosamide inhibits the proliferation of cancer cells in colon (Sack et al. 2011), breast (Londono-Joshi et al. 2014), prostate (Lu et al. 2011), and lung cancers (Stewart et al. 2016), as well as GBM (Wieland et al. 2013). Moreover, niclosamide enhanced the anti-proliferative activities of oxaliplatin in human colorectal cancer (Osada et al. 2011) and of cisplatin in lung cancer (Zuo et al. 2018). Wieland et al. also reported that niclosamide acts as a natural inducer of NFKBIA and combined treatment of niclosamide and TMZ synergistically inhibited cellular viability in NFKBIA ± glioblastoma genotype (Wieland et al. 2013). Our results are consistent with those of previous studies, showing the anti-proliferative activities of niclosamide as well as synergistic effects of combined treatments.

In the case of GBM, the cell of origin is in the subventricular zone (Yoon et al. 2020; Lee et al. 2018), but the cells in which the tumor is present are a realistic therapeutic target. Cancer stem cells (CSCs) have been tested as targets of therapeutic agents because such cells are resistant to chemotherapy (Dean et al. 2005). In large tumors, however, cancer cells may acquire or lose stemness, resulting in tumors containing large numbers of CSCs (Kang et al. 2015). We therefore tested the effects of niclosamide and/or temozolomide on heterogenous GBM TSs. Some studies reported that niclosamide reduces the stemness of CSCs in breast cancer (Wang et al. 2013) and chronic myelogenous leukemia (Jin et al. 2017) by inhibiting the Wnt/*β*-catenin pathway. Similarly, it was confirmed that GBM cells pre-exposed to niclosamide had decreased stemness in neurosphere assays (Wieland et al. 2013). Consistent with previous results, we found that combined treatment with niclosamide and temozolomide significantly reduced the stemness of GBM TSs, with western blotting showing that this combination reduced the expression of stemness markers.

The EMT has been implicated in cancer progression, and the canonical Wnt signaling cascade has been found to control transcription factor Snail, which triggers EMT in human cancer by suppressing the expression of epithelial cell genes (Barrallo-Gimeno and Nieto 2005). Niclosamide is known for a potent inhibitor of Wnt/*β*-catenin signaling in various types of cancer (Sack et al. 2011; Wieland et al. 2013). Ahn et al. (2017) revealed that niclosamide directly targets Axin-GSK3 interactions, suppressing Wnt/Snail-mediated EMT in human colon cancer. Some studies reported that niclosamide significantly reduced invasiveness of breast (Ye et al. 2014) and lung (Stewart et al. 2016) cancer cells. However, the ability of the combination of niclosamide and temozolomide to inhibit invasiveness of GBM TSs had not yet been reported. We confirmed for the first time that the combination treatment of niclosamide and TMZ effectively reduced the invasiveness of GBM TSs, suppressing Snail mediated EMT.

Temozolomide, known as alkylating agent, makes DNA methylation of guanine at O^6^ position, leading to double strand break and induction of cancer cell apoptosis (Trivedi et al. 2005). Because DNA repair enzyme O6-methylguanine-DNA methyltransferase (MGMT) removes this methylation (Kokkinakis et al. 2001), the methylation extent of the MGMT promoter has been regarded as a prognostic factor for GBM (Hegi et al. 2005). Kohsaka et al. (2012) found that STAT3 inhibition downregulated MGMT expression and overcame temozolomide resistance in GBM cell lines. Because it is well known that niclosamide acts as a STAT3 pathway inhibitor (Ren et al. 2010; Gyamfi et al. 2019), we conducted immunoblotting and found that the combination treatment of niclosamide and temozolomide suppresses the expression of phosphorylated STAT3 as well as MGMT. This result suggests that STAT3 inhibition could be a potential synergistic mechanism of the combination treatment.

Transcriptome analyses comparing expression profiles before and after combined treatment of niclosamide and TMZ revealed that a subset of genes related to stemness and EMT were significantly downregulated. Gene Set Enrichment Analysis (GSEA) also showed that autophagy related gene set was upregulated after combined treatment, suggesting that the combination treatment has anti-proliferation effect as well as anti-stemness and anti-invasiveness effects.

Several orthotopic xenograft models have shown that niclosamide inhibits tumor growth and metastases in various types of cancers (Osada et al. 2011; Li et al. 2013). One glioblastoma study also reported that niclosamide-pretreated group had survival benefit compared with untreated group in a mouse orthotopic xenograft model (Wieland et al. 2013). However, the therapeutic potential of combination treatment of niclosamide and TMZ against GBM TSs in vivo is still unknown. Here, we found that the combination treatment of niclosamide and TMZ significantly prolonged survival period in our experiments. These results could be explained by RNA sequencing and immunoblotting data that niclosamide treatment might have impact on the cancer signaling cascades which are associated with stemness, invasiveness, and proliferation.

In summary, the combination of niclosamide and temozolomide effectively reduced the viability, stemness and invasion capacity of GBM TSs as well as prolonged survival period in a mouse orthotopic xenograft models, suggesting that this combination could be treatment option for GBM patients in clinical setting.

## Electronic supplementary material

Below is the link to the electronic supplementary material.

Supplementary file1 (PDF 332 kb)
