# Supplementary material for: Combined effects of niclosamide and temozolomide against human glioblastoma tumorspheres
Source: J Cancer Res Clin Oncol. 2020 Jul 25;146(11):2817–28. doi: 10.1007/s00432-020-03330-7 (PMC7519913; doi:10.1007/s00432-020-03330-7)
Supplement: Supplementary file 1 — Supplementary file1 (PDF 332 kb) [file 432_2020_3330_MOESM1_ESM.pdf]

Combined effects of niclosamide and temozolomide against human glioblastoma tumorspheres

Hyeong Cheol Oh[1], Jin-Kyoung Shim[1], Junseong Park[1], Ji-Hyun Lee[1], Ran Joo Choi[1], Nam Hee Kim[2], Hyun Sil Kim[2], Ju Hyung Moon[1], Eui Hyun Kim[1], Jong Hee Chang[1], Jong In Yook[2], Seok-Gu Kang[1*]

[1]Department of Neurosurgery, Brain Tumor Center, Severance Hospital, Yonsei University College of Medicine, 50-1 Yonsei-ro, Seodaemun-gu, Seoul 120-752, Republic of Korea

[2]Department of Oral Pathology, Yonsei University College of Dentistry, 50-1 Yonsei-ro, Seodaemun-gu, Seoul 120-752, Republic of Korea

*Corresponding Author

Seok-Gu Kang, M.D., Ph.D. (ORCID: 0000-0001-5676-2037), Department of Neurosurgery, Brain Tumor Center, Severance Hospital, Yonsei University College of Medicine, 50-1 Yonsei-ro, Seodaemun-gu, Seoul 03722, Republic of Korea. Tel: +82-2-2228-0882; Fax: +82-2-393-9979; E-mail: seokgu9@gmail.com

Jong-In Yook, D.S., Ph.D., Department of Oral pathology, Yonsei University College of Dentistry, 50-1 Yonsei-ro, Seodaemun-gu, Seoul 03722, Republic of Korea. Tel: +82-2-2228-3032; Fax: +82-2-393-9979; E-mail: jiyook@yuhs.ac

Supplementary data

Supplementary Materials and Methods

Methods are described more detailed in the following section:

RNA QC, library construction, and sequencing

The quality and quantity of total RNA were assessed by Agilent 2100 Bioanalyzer with a Eukaryotic Total RNA Pico chip (Agilent Technologies). Using this instrument, the 18s/28s ratio and RNA Integrity Number (RIN) values were determined from the Bioanalyzer traces. 100ng of non-FFPE RNA from each sample was chemically fragmented to a size appropriate for library preparation. The RNA fragments were bound to random primers and synthesized to strand specific cDNA with Actinomycin D solution. 3' end A tailing and adaptor-ligated cDNA were amplified for 14 cycles. The samples were concentrated to 200 ng in 3.4 µL DW using a Speedvac machine (Thermo Scientific) and hybridized with All Human V6+UTR baits at 65°C for 16 h. After hybridization, the biotinylated libraries were captured by M-270 streptavidin beads (Thermo Fisher Scientific). PCR was performed for 12 cycles to amplify the captured libraries and add the unique index tags. Libraries were quantified using the Agilent TapeStation 4200 HSD1000 screen tapes (Agilent Technologies) and KAPA Library Quantification Kit (KK4824, Kapa Biosystems). The individual samples were pooled and sequenced on the Illumina NovaSeq6000 with 150 bp paired-end by following the manufacturer's protocols. Image analysis were performed using the NovaSeq6000 control Software version 1.3.1 and the output data was demultiplexed with bcl2fastq v2.2 generating fastqc files.

Supplementary figures legends:

Supplementary Figures

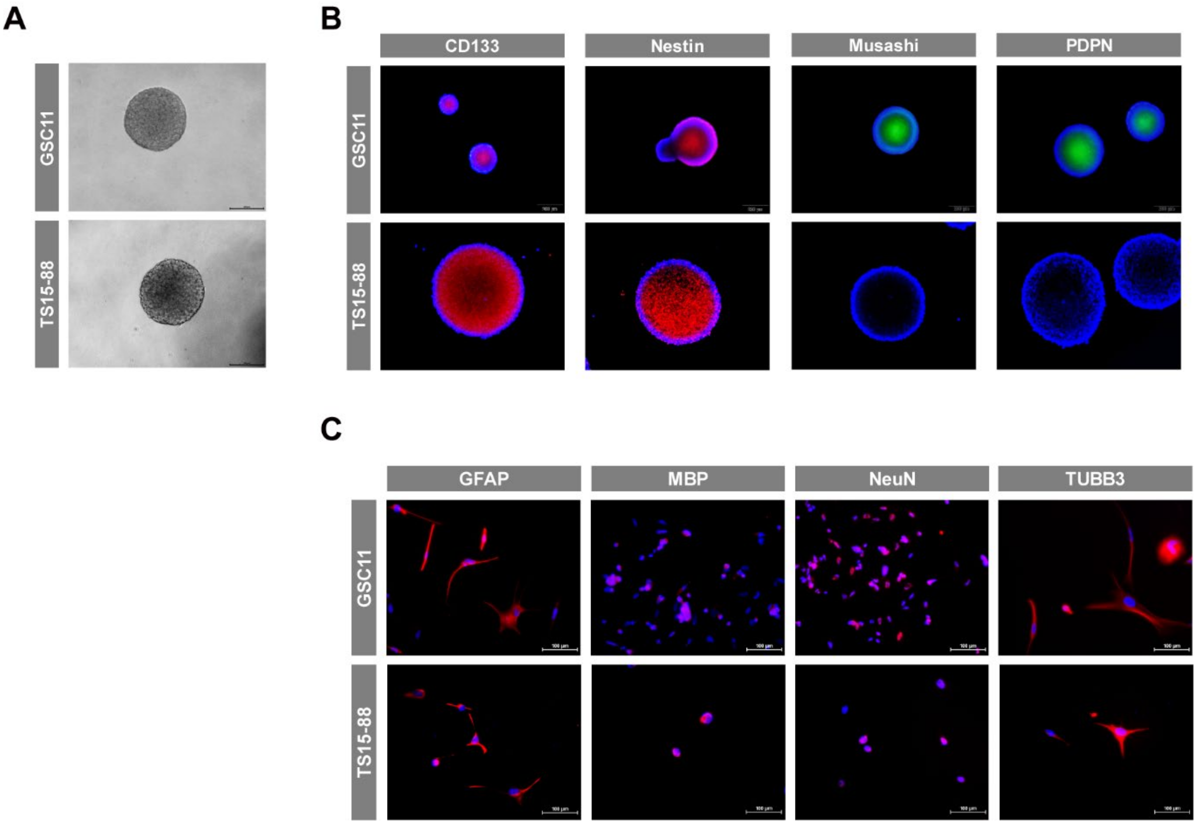

Supplementary Fig. 1 Characterization of GBM TSs. **a** TS formation was observed after 3 weeks of culture in GSC11 and TS 15-88 sphere. GSC11 and TS15-88 showing expression of markers for **b** stemness and **c** neuroglial differentiation. Images are ×100 original magnification with scale bar = 50μm for **a** and **b**, ×200 original magnification with scale bar = 200μm for **c**. *GFAP, glial fibrillary acidic protein; MBP, myelin basic protein; PDPN, podoplanin; TUBB3, tubulin beta-3 chain*

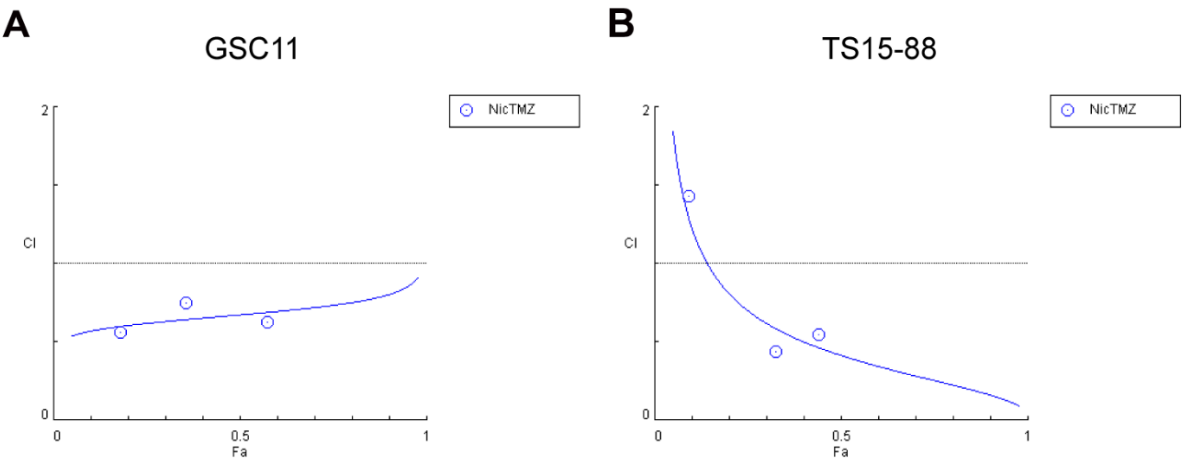

Supplementary Fig. 2 Combination indices (CIs) of the combination treatment of niclosamide and temozolomide, calculated by CompuSyn software. The blue marks in the middle, both in graph A and B, indicate the results of combination treatment of niclosamide (500 nM) and temozolomide (250 µM) that is used in the experiment. a, b both graphs showed CI values lower than 1, suggesting synergism of the combination treatment.
